# The management of COVID-19 cases through telemedicine in Brazil

**DOI:** 10.1371/journal.pone.0254339

**Published:** 2021-07-14

**Authors:** Alfredo Montelongo, João Luiz Becker, Rudi Roman, Elise Botteselle de Oliveira, Roberto Nunes Umpierre, Marcelo Rodrigues Gonçalves, Rodolfo Silva, Katarzyna Doniec, Ali K. Yetisen

**Affiliations:** 1 TelessaudeRS, Department of Epidemiology, School of Medicine, UFRGS, Porto Alegre, Brazil; 2 Department of Operations Research, Universidade Federal do Rio Grande do Sul (EA/UFRGS), Porto Alegre, Brazil; 3 Management School, Fundação Getulio Vargas (FGV/EASP), Rio de Janeiro, Brazil; 4 School of Medicine, The Federal University of Rio Grande do Sul, Porto Alegre, Brazil; 5 Department of Sociology, University of Cambridge, Cambridge, United Kingdom; 6 Department of Chemical Engineering, Imperial College London, South Kensington Campus, London, United Kingdom; University of South Australia, AUSTRALIA

## Abstract

In Dec 2020 Brazil became one of the worldwide epicenters of the COVID-19 pandemic with more than 7.2M reported cases. Brazil has a large territory with unequal distribution of healthcare resources including physicians. Resource limitation has been one of the main factors hampering Brazil’s response to the COVID-19 crisis. Telemedicine has been an effective approach for COVID-19 management as it allows to reduce the risk of cross-contamination and provides support to remote rural locations. Here we present the analyses of teleconsultations from a countrywide telemedicine service (TelessáudeRS-UFRGS, TRS), that provides physician-to-physician remote support during the COVID-19 pandemic in Brazil. We performed a descriptive analysis of the teleconsultation incoming calls and a text analysis from the call transcripts. Our findings indicate that TRS teleconsultations in Brazil experienced an exponential increment of 802.% during a period of 6 days, after the first death due to COVID-19 was reported. However, the number of teleconsultations cases decreased over time, despite the number of reported COVID-19 cases continuously increasing. The results also showed that physicians in low-income municipalities, based on GDP per capita, are less likely to consult the telemedicine service despite facing higher rates of COVID-19 cases. The text analysis of call transcripts from medical teleconsultations showed that the main concern of physicians were “asymptomatic” patients. We suggest an immediate reinforcement of telehealth services in the regions of lower income as a strategy to support COVID-19 management.

## Introduction

During the 2020 COVID-19 pandemic Brazil became one of the worst hit countries with (7’040,608) confirmed cases and (183,735) deaths as of 17^th^ of December 2020 [1]. Brazil has large regional disparities in access to health services and unequal distribution of physicians, which constitutes a considerable challenge in meeting the diagnostics and treatment needs during the COVID-19 pandemic. For example, Amapá, which is a low income state (by GDP per capita), has 0.44 physicians per 1000 people while Espirito Santo 10.41 physicians. Physicians attending in remote municipalities experience high patient turnovers, moreover the lack of specialists makes referrals and consultations of non-standard cases impractical. Telemedicine is an effective approach to support clinical practitioners based in remote locations to manage COVID-19 patients [2–4].

**Fig 1:**
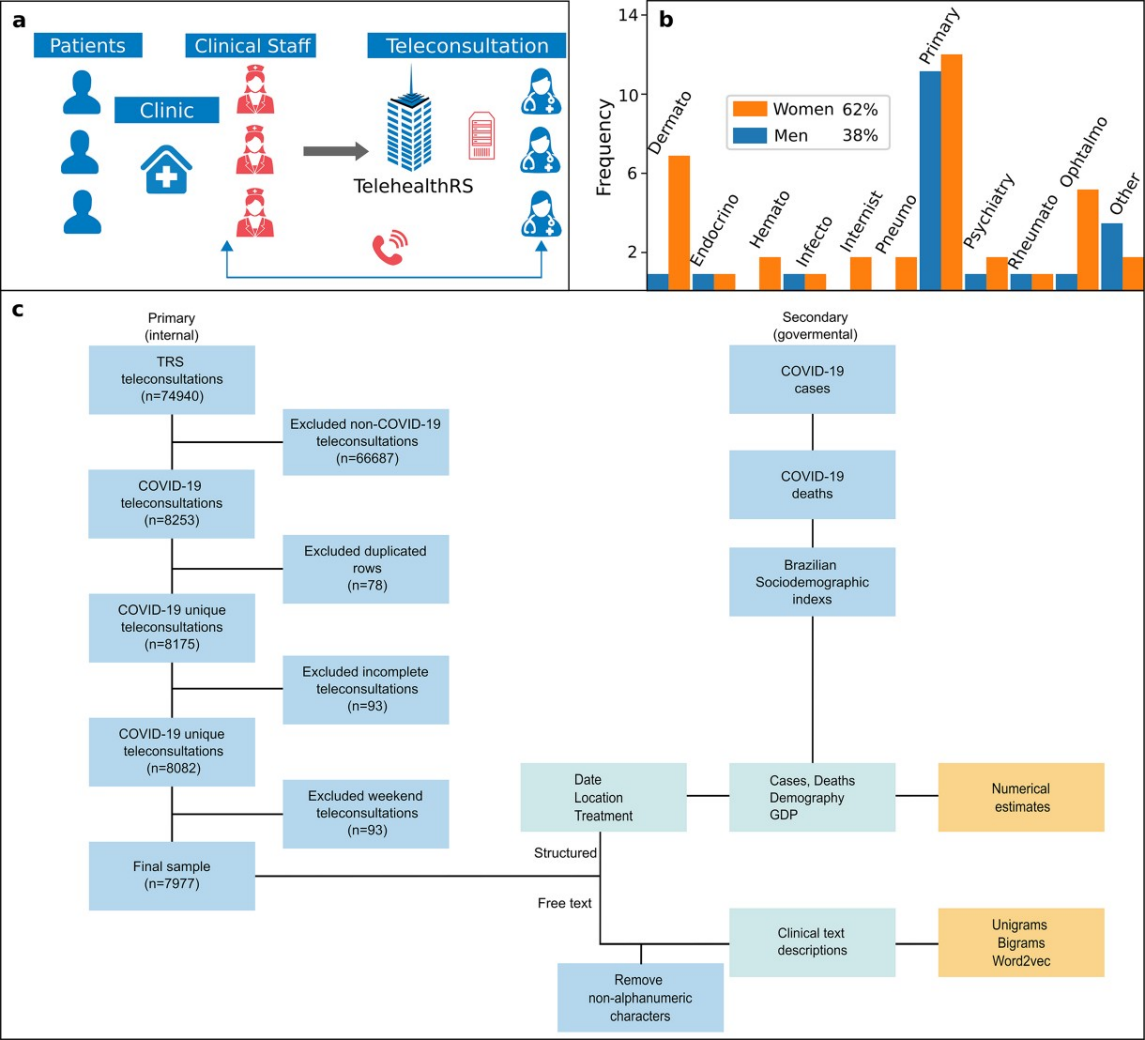
Structure of TRS and the distribution of teleconsultations in Brazil. **(a)** A flow process to perform teleconsultations at TRS, medical staff from clinical centers contact TRS through a telephone line (0800) that is answered by a physician teleconsultant **(b)** The frequency of physicians at TRS by specialization and gender **(c)** The flowchart of the process to retrieve and process the data for this study. The data was retrieved from primary (TRS) sources that passed through a validation process and secondary sources. Finally the free text data was preprocessed and used to estimate frequencies and vector representations and the structured text and secondary sources to plot the rest of the information.

TRS is a free of charge telehealth service funded by The Brazilian Ministry of Health which offers services of teleconsulting, telediagnosis, and distance learning to publicly medical units. TRS was established in 2007 as a research project of Federal University of Rio Grande do Sul (UFRGS) medical school [5–8]. Although TRS offers a broad scope of services, the present work will focus on teleconsultations for health professionals, doctors and nurses, from more than 40,000 medical centers across Brazil, particularly in distant locations. Teleconsultations for health professionals are used in two main contexts: (i) to discuss non-standard patient cases and (ii) to inquire about medical practices (e.g. medical procedures). **Fig 1A** shows the processes that TRS follows to perform a remote medical teleconsultation. Health workers from the clinic units contact TRS through a toll-free number (0800), where they can discuss specific patient doubts or clear medical practice questions, telehealth consultants retrieve information looking up on specialized medical databases (e.g. DynaMed) or through medical group discussions within physician colleagues from TRS. Operations of TRS are located in a complex housed in a 13,000 ft^2^ facility in Porto Alegre Brazil, where it employs 160 professionals, including 82 physicians from diverse array of specialties. During the COVID-19 crises, most of the TRS personnel began working from home using a VPN service. The personnel who continued working in TRS location respected social distancing rules. **Fig 1B** shows the gender composition of staff members, in which the majority are female (62%), including the number of specialists per discipline.

Telemedicine and the interchangeable term telehealth is a denomination of a medical activity that involves an element of distance and an electronic communication [9, 10]. Conventional forms of telemedicine include clinician-to-patient, patient-to-mobile and patient-to-patient interactions. Even though telemedicine already provided advantages to health care systems [10], the COVID-19 pandemic increased demand for remote consultations. For example, earlier in 2020 the number of consults in the USA via telemedicine was 0.1% of the total number of consultations and by April 2020 this number increased to 69% [11].

Previous studies on telemedicine during the COVID-19 pandemic have focused on applications that include, forward triage [12], automated logic flows (bots) [12], management of chronic conditions [13], mental health services [14], palliative care [15] and teleneurology [16]; challenges, that include, coordination and integration of involved sectors [12, 17, 18], creation of regulatory frameworks [4, 13, 17–19], accessibility to mobile technologies [20]; reviews [21, 22]; suggestions of a Software-Defined Network architecture for telemedicine purposes [23]; suggestions on potential benefits of using telemedicine for vulnerable groups that do not have access to clinical services due to COVID-19, such as gestant women from developing countries, as pregnancy has been reported as a clinical condition with risk factors [24, 25]; discussions on the legislation of telemedicine as its adoption has become increasingly widespread and new legislation frameworks will need to be developed to satisfy this new demand [26]; discussions about lessons of operationalizing telemedicine in ophthalmology and its potential effect for health systems [27, 28]; propositions of models of clinical attention that consisted in a mix of in-person visits with telemedicine support [29]; discussions of solutions for long-term COVID-19 consequences that can include telemedicine support [30]; propositions of offering telehealth service in public spaces as a form to prevent the intimated partner violence (IPV), as it has been reported that this situation has been increased during the pandemic COVID-19 due to the process of lockdown [31]; studies of the impact of COVID-19 of clinical trials which concluded that the pandemic has damaged the quality of the studies [32].

Up to date, the published studies have been focused on physician-to-patient interactions [3], the objective of this work is to present an empirical analysis of a physician-to-physician teleconsultation support service provided by TRS to medical center staff during the COVID-19 pandemic in Brazil. This study analyzed (i) geographical distribution of the COVID-19 call teleconsultations, (ii) the number of confirmed cases and deaths due COVID-19, trends in the demand for teleconsultations, (iii) and the content analysis of the topics mentioned by attending physicians during teleconsultations calls. The rest of this paper is structured as follows: methodology, results, discussions, implications of practice and future research.

## Methodology

This work was carried out in TelessáudeRS-UFRGS (TRS). We received approval to access fully anonymized data by Ethics Committee from “Hospital de Clínicas de Porto Alegre, RS” (AGHUse 2007–0402 and CAAE: 69727517.0.0000.5327). We ensured that the analyses did not compromise the identity of the patients and physicians before accessing them. The text data and phone calls analyzed did not contain any identifying information, neither from the patients nor the physicians.

### Data collection

We performed a descriptive analysis of COVID-19-related teleconsultations data, dating from the first received call related to COVID-19 in TRS, (29^th^ February 2020), to (27^th^ November 2020). **Fig 1C** shows a flow chart of the process that we followed to perform the analyses. Our data was retrieved from two sources, primary (internal) from TRS which included the transcripts of teleconsultations available in free text form that were typed by each attending physician during the teleconsultations with their corresponding information (date, location, suggested treatment) for each of the calls and (n = 7977 COVID-19 teleconsultations); and secondary (external) from Brazilian government sites (no. of COVID-19 cases, no. of COVID-19 deaths, demography and GDP). To select the internal data from TRS we filtered the calls that corresponded to the COVID-19 teleconsultations, that were classified either by the user of the service or the teleconsultant (physicians), during each of the calls, to ensure the robustness of the data, structure of the database and text formatting, we performed a visual inspection from a sample of cases chosen at random. From the writing of the texts, we did not identify major issues. From the structure of the database, we identified duplicated cells that we removed; non concluded teleconsultations, as some of them require to consult extra information and are marked with this status in the database, we removed non-concluded teleconsultations and finally we filtered the teleconsultations performed during weekdays because TRS only work during these days, the ones that appeared in weekends were accessed by teleconsultants out of labour days. From the formatting aspects we identified that the text contained html tags symbols, such as <BR>. To deal with these issues, we performed a pre-processing of the text by removing the html tags. We also retrieved COVID-19 morbidity and mortality data from Brazilian Ministry of Health [1, 33], and socioeconomic indexes Gross Domestic Product (GDP) and population from Brazilian Institute of Geography and Statistics (IBGE) [34].

### Data analysis

We performed a geographical analysis of incoming COVID-19 calls. The study utilized a division of the country based on the 5 regions (North, North-E, Central-W, South-E, South) designed by IBGE. We analyzed the number of teleconsultations and the rate of reported cases by region, we estimated the rate by dividing the reported COVID-19 cases into the population of each region. This involved identifying the origin of calls (municipalities), studying the percentage of municipalities that TRS have attended in the region and their corresponding GDP per capita. Additionally, we analyzed how the number of confirmed cases and deaths due COVID-19 affected call teleconsultations over time. Therefore, this study integrated the TRS and Ministry of Health data during COVID-19 pandemic. The suggested treatment for teleconsultations were studied over the time, and time teleconsultation duration with their corresponding descriptive statistics (mean, sd, interquartile).We divided the data into three categories based on a visual inspection from interquartile ranges: (i) conventional teleconsultations for typical cases (1–20 mins) within the 1^st^ and 3^rd^ box plot quartile, (ii) teleconsultations with increasing time usually requiring medical discussions with colleagues from other specializaties (20–42 mins)—higher than 3rd quartile, and (iii) atypical teleconsultations (42 min- or more), that refers to multiple teleconsultations in a single call.

Call transcripts from medical teleconsultations were written in the form of free text, to identify the age of the patients we designed an algorithm that performed a regex string matching search of the term “years old” saved its precedence number(s) into a structured form and validated the results by sampling some of the texts. As we wanted to understand the age distribution of the patients discussed on the teleconsultations according to the demographic Brazilian distribution, we divided the age of the patients by the Brazilian demographic distribution and estimated a rate index.

As the nature of some of our data was in the form of text, we searched for alternatives to transform the qualitative information into a quantitative representation. We identified two strategies, the first one was to estimate the frequency of terms that provided the most common mentioned terms and the second one estimating clusters of topics. To calculate the frequency of terms, we first performed a pre-processing of the texts by transforming into lowercase, removing non-alphabet symbols and stop words, frequently used words that do not have a qualitative meaning () such as “a”, “the”, “but”, using the library “word cloud” [35] from Python software. We estimated the frequency of words in two forms: unigrams (unique words) and bigrams (two words together), and selected the most frequent words that we considered with useful meaning to COVID-19. The second strategy was to use a Natural Language Processing (NLP) technique to identify clusters of words that represented a topic. We utilized the word2vec [36] methodology that transforms each word into a vector space representation and enables us to identify terms that appear together (clusters). To perform this word2vec representation we used the software Gensim [37], also using a pre-processing process in the same way as the frequency. We used the default parameters (size = 100, window = 5 and min_count = 500), where size refers to the length of the vector, window refers to the number of words taken into account along each of the words and min_count refers to the minimal number of words to take into account. Finally, we plot the data of the model using the technique t-distributed stochastic neighbor embedding (t-SNE) that represents multidimensional vectors in a 2D plot, based on the proximity of the terms. We used the software sklearn [38] using the parameters (perplexity = 3, n_components = 2, init = ’pca’ and, n_iter = 2500) suggested from [39], where perplexity defines how to balance attention between local and global aspects of the data, n_components defines the number of dimensions to be plot, init defines the initialization method and n_iter defines the number of iterations.

## Results

### Teleconsultations during the COVID-19 pandemic in Brazil

**Fig 2:**
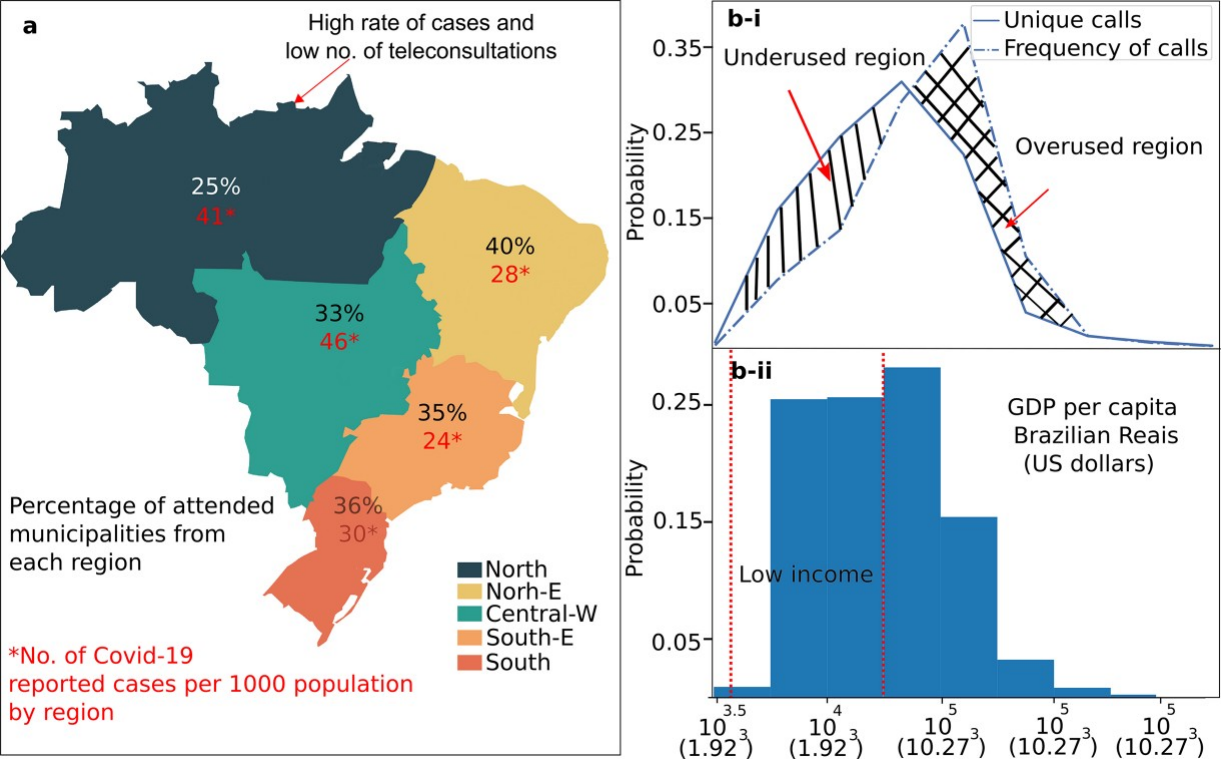
The proportion of municipalities attended by TRS in Brazil by region. **(a)** The Northern region has a high rate of confirmed cases (41) but a low number of teleconsultations (25%). **(b)** The percentage of attended municipalities according to its GDP per capita. The straight line represents the frequency of calls by GDP (unique calls), while the dashed line is the frequency of municipalities taking all calls into consideration. High-income municipalities perform more COVID-19 teleconsultations than low-income municipalities, the differences denote overused and sub used regions.

Since the first reported case of COVID-19 in Brazil (27^th^ February 2020), TRS has assisted 7977 COVID-19 related calls from 1527 cities and towns. **Fig 2A** illustrates the distribution of towns that have been attended by TRS and the number of COVID-19 related calls per each region as of 27^th^ November 2020. The region with the highest rate of reported COVID-19 cases per 1000 population, according to the official data from the Ministry of Health was the Central-W (46) (CW) (46). The second region was the Northern (N) (41) region, where the Amazon forest is located. This region has also the second lowest rate of call teleconsultations (25%). Moreover, this region has the lowest income in Brazil based on Gross Domestic Product (GDP) per capita (5% of the total GDP). The percentage of attended municipalities located in Northeast (NE), Southeast (SE) and the South (S) regions were 28%, 24% and 30% respectively.

**Fig 2Bi** shows the Brazilian GDP per capita by municipality, which follows a left skewed distribution common for developing countries, most of the towns have a low income. **Fig 2Bii** shows the relationship between municipalities measured by GDP per capita (i) coverage by TRS (at least one call since the first confirmed case in Brazil) and (ii) frequency of calls from these municipalities (dashed line). We differentiated these two measures to estimate under and overused service according to GDP per capita by town. Thus, a hypothetical balance measure will be that the frequency of calls will be equally plotted among both graphs, however municipalities with lower income had less teleconsultations (underused regions) than municipalities with higher incomes (overused regions).

**Fig 3:**
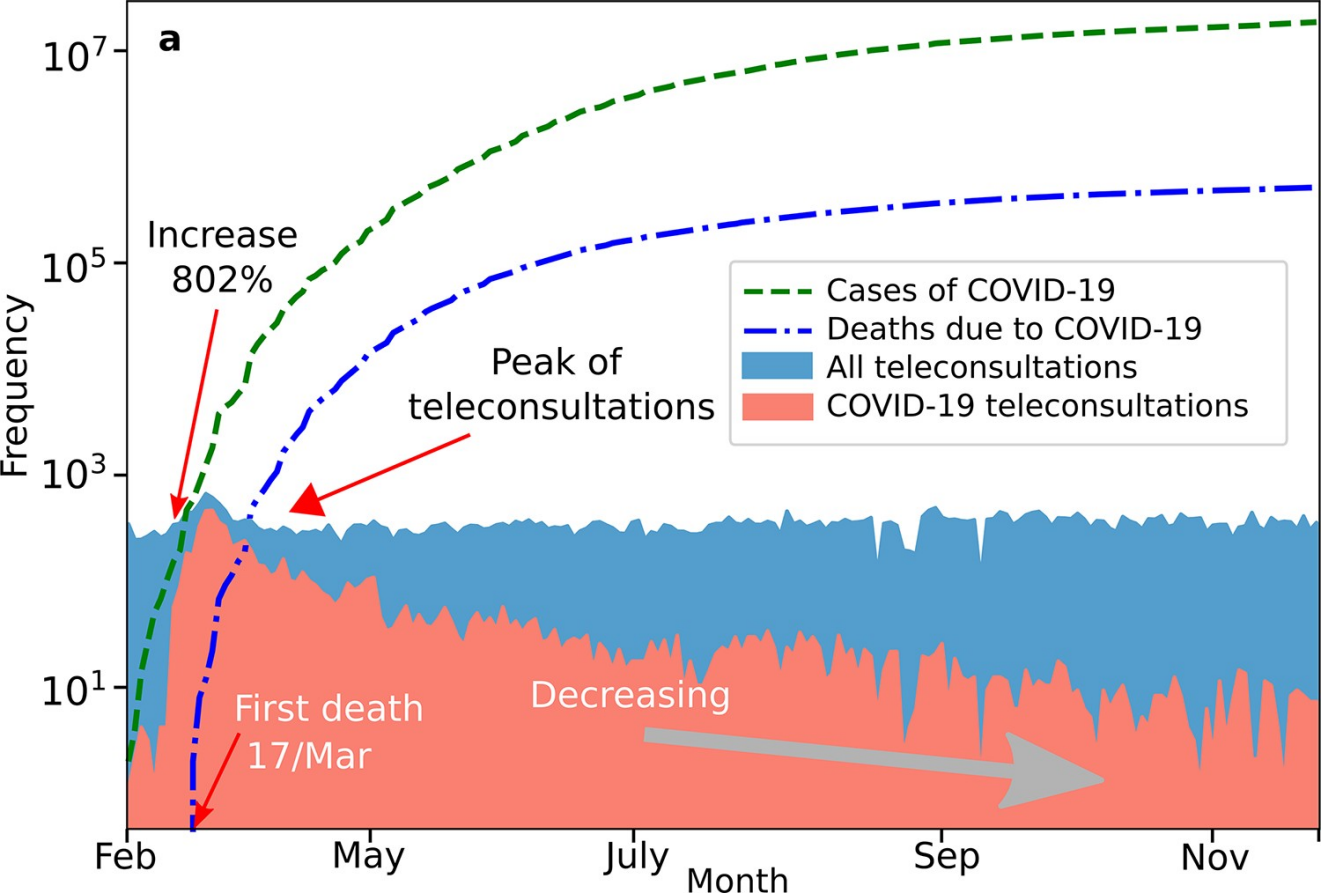
(a) The number of teleconsultations of COVID-19 and non-COVID 19 over time. The number of teleconsultations followed an exponential increment until the first reported death case on 17 March 2020, however they started decreasing despite the fact that the number of COVID-19 cases and deaths continued increasing.

We examined temporal changes in demand for COVID-19 and all teleconsultations, compared to the number of COVID-19 reported cases and deaths in Brazil. **Fig 3** shows the frequency of events since the first COVID-19 case was confirmed in Brazil, on (27^th^ February 2020), until the last day of our database on 27 Nov 2020. We visually categorize three phases of pandemic: (i) the starting period—that takes 14 days (27 February—12 March 2020), (ii) the exponential increase period that takes around 8 days (12 March—19 March/2020) and (iii) decreasing period (20 March—27 Nov 2020). During the exponential phase, the number of COVID-19 teleconsultations increased 802% during the first 6 days, from 56 teleconsultations on 12 March to 449 on 19 March 2019. Unexpectedly, the number of teleconsultations of COVID-19 decreased after the exponential increasing period despite the number of COVID-19 cases and deaths continuing increasing.

**Fig 4:**
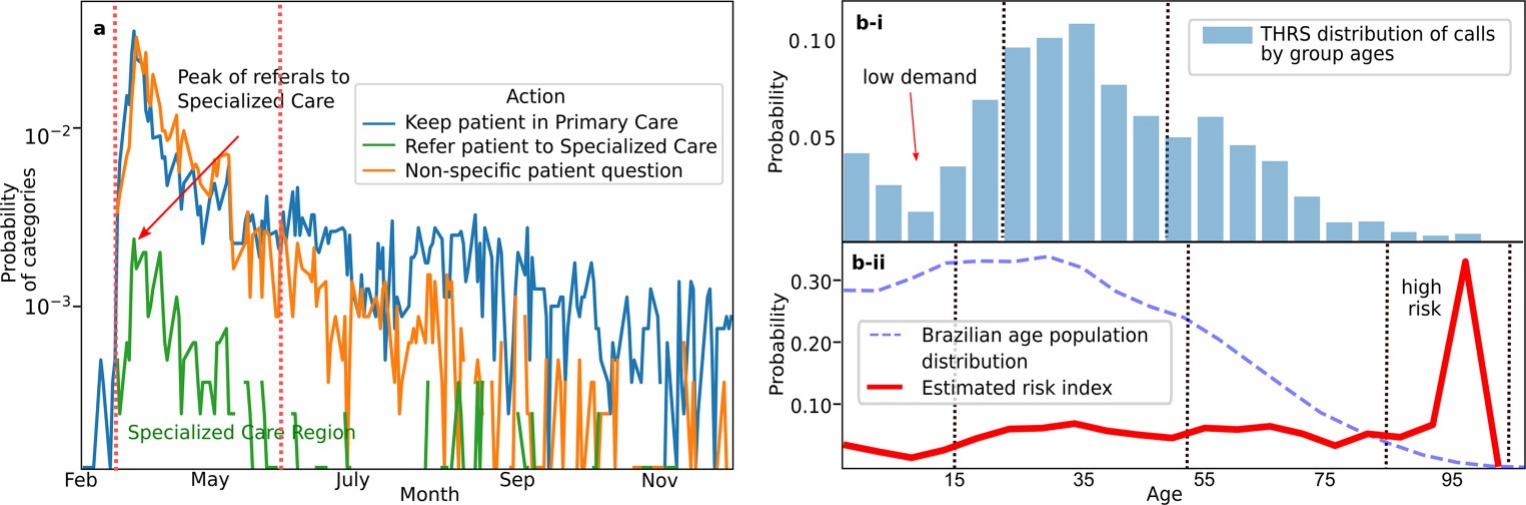
Telemedicine calls over the time with resolutions and patients age teleconsultations. **(a)** The suggested actions on teleconsultations are divided into: maintaining patient in primary care (observation/isolation) or sending patient to emergency care attention such as Intensive Care Unit (ICU), the rest of the teleconsultations are general practice inquiries. **(b)** The distribution of patients’ age discussed on teleconsultations and its relation to the Brazilian demographic population. The red line represents a rate between patients ages teleconsultations and Brazilian demographic distribution, the chances of a physician from a medical center to call and discuss patients over 80 years are 5.0 times bigger than the rest of the groups.

**Fig 5:**
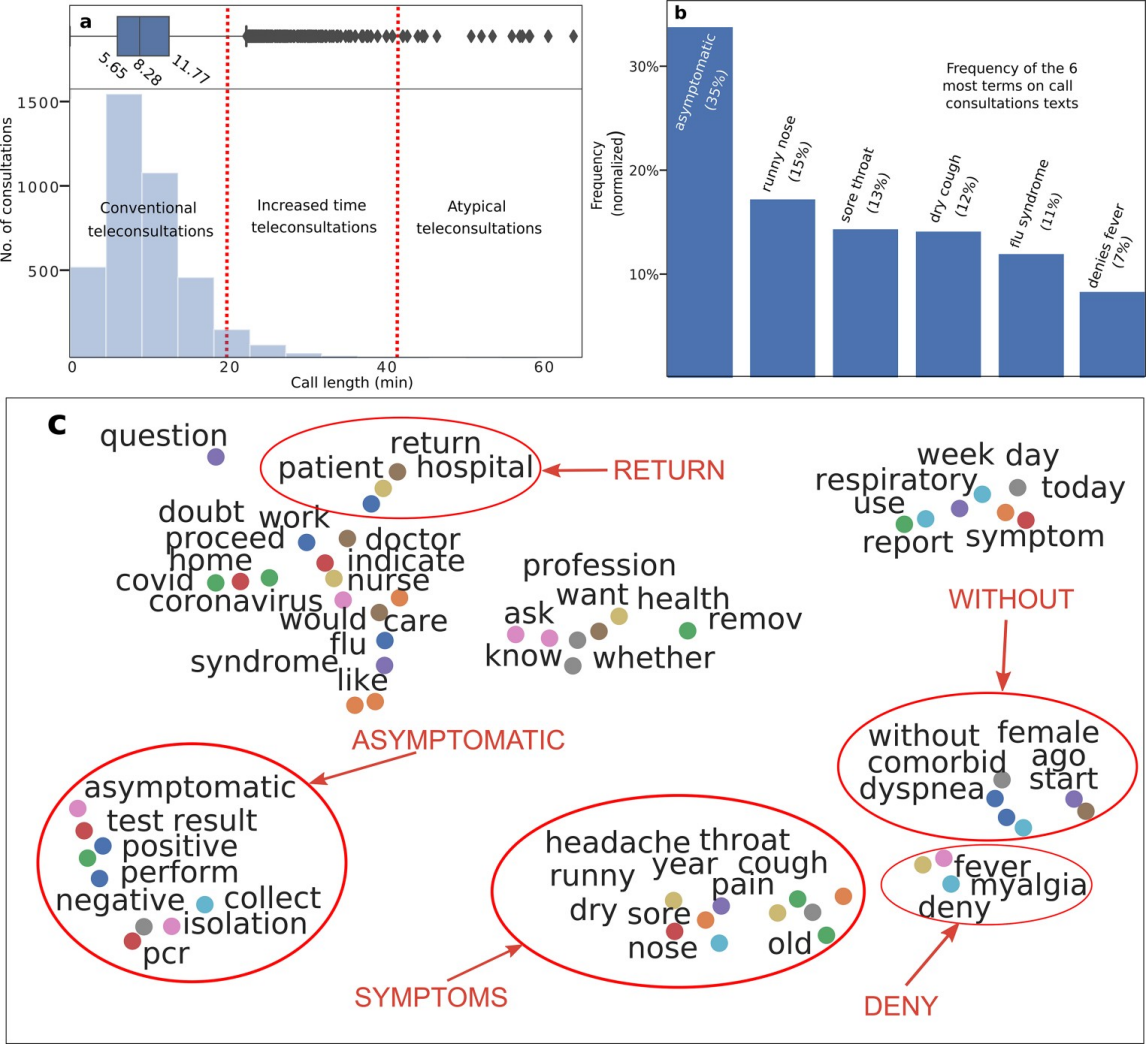
Frequency and relationship of words within annotated texts from teleconsultations. **(a)** The time length of the call in an histogram and boxplot, that are divided into three groups: conventional, atypical and long. **(b)** The 6 most frequent terms in teleconsultations. The most frequent term is “asymptomatic” (35%), followed by runny nose (15%), sore throat (13%), dry caught (12%), flu syndrome (11%) and denies fever (7%). **(c)** Relations of words. Closer distances imply that terms were used together. We categorized 4 topics: suspect, asymptomatic, symptoms and doubts. The plot increases evidence on physician concerns about asymptomatic patients, as the symptoms topic appears together with the word “without”.

**Fig 4A** shows the teleconsultation results divided into two categories according to TRS policies: (i) refer the patients to a specialized care, such as centralized hospital or emergency department (green region) (9%), (ii) maintain patient under observation/isolation (91%) (blue region), which represents mild medical, oligosymptomatic or asymptomatic patients, the rest refers to general practice inquiries about COVID-19. **Fig 4B-i** illustrates age data from patients teleconsultations, where values between 20 and 50 years are the most discussed. **Fig 4B-ii** shows an index “probability demographic group call” based on the rate between the age of the patient’s teleconsultations and Brazilian demographic population. Therefore, the probability of a physician to use THRS, to discuss a patient that belongs to the group, older than 80 years (high risk)—are 5.0 times bigger (0.41), than for a non-risk group (0.08), less than 10 years old. It can be concluded that physicians are less concerned about discussing COVID-19 cases in children. We show in **Fig 5A** the time length of teleconsultations that we classified into three groups as: conventional (96.3%)—less than 20 mins, increased time (3.59%) between 20 and 40 mins, and atypical teleconsultations (1%)—longer than 40 mins and usually requires external knowledge.

Finally we analyzed the content of teleconsultation notes in order to understand the most frequent topics discussed during the telemedicine calls. **Fig 5B** illustrates the six most frequent terms and their frequencies. ‘Asymptomatic’ was the most frequent term (35%), which showed that the healthcare staff was particularly concerned with cases which did not present symptoms [40]. Interestingly, the second and third most frequent terms were runny nose (rhinorrhea) (15%) and sore throat (13%), however they have a prevalence of less than 5% in reported studies [33, 41], what indicates that physicians are concerned with symptoms which are not well defined by studies. **Fig 5C** shows distances between linguistic terms. The closeness of the words represent their correlational proximity in a teleconsultation context. We detailed 5 clusters: return, which shows that physicians are concerned for patients that returned to the “hospital”; asymptomatic, which is together with “test”, “positive”, “negative” and “pcr”, which suggests that physicians have doubts about asymptomatic patients, that test either positive or negative; symptoms, that shows “headache”, “sore throat”, “dry cough” and “runny nose”. Both, asymptomatic and symptoms, are in line with results of frequency terms detailed on **Fig 5B** without, which appears close to “dyspnea” and “female”; and deny that contains the terms “fever” and “myalgia”. We did not identify symptoms related to other non-COVID-19 conditions.

## Discussion

Our results show that physicians based in towns with lower GDP per capita and with higher number of reported cases are less likely to use the service. A plausible explanation is the *Inverse Care Law* which states that “the availability of good medical care tends to vary inversely with the need for it in the population served” [42]. The number of teleconsultations increased 802.8%, in the course of 6 days during the first stage of the pandemic, in this context we discovered a counterintuitive result from the analyses, even though the number of COVID-19 cases and deaths continued increasing in the country, the demand for COVID-19 teleconsultations decreased along the time. This may be the result that physicians acquired enough knowledge to manage COVID-19 patients independently as well as that the available medical literature devoted to COVID-19 increased exponentially, on January 2020 the number of publications were less than 100, while in May 2020 were more than 2000 [43]. Our results of TRS actions on how to treat COVID-19 patients were estimated as 9% of send to specialized care, that is closely correlated to Brazil reported data on the number of patients referred to ICU, between 3% and 12% [44], but there were notable country differences in rates of COVID-19 cases requiring ICU admission to other countries, eg, Italy (12%) [45] vs China’s (5%) [46]; however [47] also cited that the rate of ICU admission has been reported with high amount of variation ranging from 3 to 100%. Regarding the time length of our teleconsultations, the mean of our data was 8.21 min that we compared to a prior study from [48] which reported the time length of physician-patient teleconsultations as 7.8 min (5.26% higher), two factors can explain such differences, the methodology of counting the time length and some of the calls of our sample included multiple teleconsultations in a single call. Regarding our text analysis, the most frequent term within the annotated teleconsultation text notes was “asymptomatic” (35.1%). A possible explanation for this finding is the lack of detailed epidemiological information on asymptomatic patients, in particular during the first stages of the pandemic [49]. This is in line with the findings from [40, 50] who cited that between 50–60% of the positive tests are asymptomatic/presymptomatic at the time of testing, which evidence that asymptomatic patients are a challenge for physicians that causes uncertainties.

The nature of our study was descriptive, therefore some limitations arise. Our conclusions are exploratory because we do not have a comparison group that allows us to perform causal inferences. We did not perform statistical analysis (eg. hypotheses testing or Bayes Factor) due to the objective as descriptive. We cannot validate the reliability of our studies in external contexts considering the lack of data from other sources. The textual data contains some areas of the country more representative, so we suggest future studies with control of confounding or extraneous variables (eg. population representation rates) to perform robust validations. Finally, the text data handles nonnumerical information, which by its nature exhibits limitations to perform quantitative analyses, for example the algorithm we used to identify the ages from patients works with a regex string matching and presuppose that the ages are written in the format “years old”. Moreover, the methodology that we used to transform the textual information into a numerical representation -word2vec- does not identify words that are out of the corpus which can lead to loss of its real meaning (semantic understanding).

## Conclusion

Our study revealed the importance of telehealth technology as a support to confront the COVID-19 pandemia. Previous studies have focused on discussing telehealth uses on physician-to-patient interactions [3]. Here we show an empirical analysis of the data collected by TRS to bring physician-to-physician support. The data showed that the population that uses the most telehealth physician-to-physician service is from high-income locations, even though populations from low incomes present higher rates of COVID-19 infection. Moreover, our information evidenced that the main concern of physicians from the front line are the asymptomatic patients.

We divided the implications of the study into theoretical and practical. For theoretical our research supported evidence to the Inverse Care Law as data suggests that the availability of good medical care vary inversely with the need for it in the population served. Moreover, we find the existence of a considerable number of doubts among physicians of asymptomatic patients. This finding could be the support for future research about the behaviour of physicians when they are challenge to treat patients that do not present clear symptoms. For practical implications the time length from our data showed that 96.3% of the consultations were cases that could be analyzed in less than 20 min, therefore, better strategies of professional medical education can be developed to identify frequent practical issues. We suggest that medical instruction about treating high-risk old patients should be reinforced, as our analysis shows that physicians are primarily concerned for this high-risk group. Public services that receive a high amount of information such as TRS can take advantage of its resources to create focused information mechanisms of conventional doubts for medical staff. For example, TRS elaborate and publish a weekly answered question of the most common consulted doubts. In addition, there may be a lack of knowledge about telehealth services in low-income locations; therefore, we suggest that advertisement of this kind of services should be strengthened into low-income locations. We also suggest that information saved in a structured format instead of free text enables more accurate quantitative analyses, therefore it will be suitable to collect medical notes in a structured format. Moreover, telehealth services can be targeted to specific groups such as pregnancy women, as studies evidenced [24] that COVID-19 have increased the risk of morbidity and death in pregnant women and neonates, and telemedicine can be a support tool to reduce the risk. There exists doubts from doctors that are repetitive, so developing systems that can automatizes this systematic process such as question answering AI systems will increase the efficiency of time response. Historically, medical education have not developed treatment programs for Telemedicine [51], therefore we suggest that medical residents be involved in telehealth programs since is training formation.

We believe that some topics can be covered for future research. The first one is the analysis of textual data on a temporal basis that will allow to identify symptoms and question patterns over time. The second one is that in house databases, as the one from TRS, can be correlated with external information to identify how particular events are affecting the physician’s doubts, eg. using Artificial Intelligence (AI) techniques of NLP to extract information from long text databases as the work from [43]. Brazilian legislation regarding telemedicine regulations was relaxed for physician-patient teleconsultation during the pandemic emergency [52], however the flexibilization of the regulations can be reversed, as medical associations historically have blocked Telemedicine practices [50]. It will be suitable to discuss how legislation will affect physician’s practice in the pos COVID-19 period. To provide research studies that evaluate the cost-effective relation for using telemedicine, in particular from developed countries such as Brazil, to provide robust evidences about the benefits of using telehealth strategies so policy-makers increase its use. In the opposite side, most of the studies have focused on the benefits of telemedicine strategies for COVID-19, however, potentially disadvantages and limitations must be in-depth discussed, eg. [32] revealed that 82.6% on a sample of 115 medical residents feel less confident of treating chronic disease patients using telemedicine therefore understanding these in-depth limitations will be suitable. The following study was focused on primary health care, therefor a study that covers secondary medical care will be suggested. Further research is needed to identify if local cultures are resistant to telehealth strategies as it has been documented that specific ethnic communities react differently from the rest of the population [53] and Brazil has indigenous territories with own habit and traditions. This study was focused on physician- to-physician interactions, however a broader study that covers physician-to-patient interaction will be suitable, in particular because legislation in Brazil has enabled remote consults since the COVID-19 pandemic started. Long-term implications of COVID-19 are little understood [30], the collected data of TRS provide information of data older than a year, therefore performing a study among the group of patients that suffered from COVID-19 at the start of the pandemic will potentially provide information about long-term COVID-19 consequences.

## Supporting information

S1 Data (CSV)

S2 Data (CSV)

S3 Data (CSV)

S4 Data (CSV)

## References

[1] Coronavírus Brasil. [cited 17 Dec 2020]. Available: https://covid.saude.gov.br/

[2] Greenhalgh T, Koh GCH, Car J. Covid-19: a remote assessment in primary care. BMJ. 2020; m1182. doi: 10.1136/bmj.m1182 32213507

[3] Ting DSW, Carin L, Dzau V, Wong TY. Digital technology and COVID-19. Nat Med. 2020 [cited 5 Apr 2020]. doi: 10.1038/s41591-020-0824-5 32284618 PMC7100489

[4] Keesara S, Jonas A, Schulman K. Covid-19 and Health Care’s Digital Revolution. N Engl J Med. 2020; NEJMp2005835. doi: 10.1056/NEJMp2005835 32240581

[5] Gonçalves MR, Umpierre RN, D’Avila OP, Katz N, Mengue SS, Siqueira ACS, et al. Expanding Primary Care Access: A Telehealth Success Story. Ann Fam Med. 2017;15: 383–383. doi: 10.1370/afm.2086 28694280 PMC5505463

[6] Katz N, Roman R, Rados DV, Oliveira EB de, Schmitz CAA, Gonçalves MR, et al. Acesso e regulação ao cuidado especializado no Rio Grande do Sul: a estratégia RegulaSUS do TelessaúdeRS-UFRGS. Ciênc saúde coletiva. 2020;25: 1389–1400. doi: 10.1590/1413-81232020254.28942019 32267440

[7] Harzheim E, Gonçalves MR, Umpierre RN, da Silva Siqueira AC, Katz N, Agostinho MR, et al. Telehealth in Rio Grande do Sul, Brazil: Bridging the Gaps. Telemedicine and e-Health. 2016;22: 938–944. doi: 10.1089/tmj.2015.0210 27096384

[8] Lutz de Araujo A, Moreira T de C, Varvaki Rados DR, Gross PB, Molina-Bastos CG, Katz N, et al. The use of telemedicine to support Brazilian primary care physicians in managing eye conditions: The TeleOftalmo Project. Csutak A, editor. PLoS ONE. 2020;15: e0231034. doi: 10.1371/journal.pone.0231034 32240268 PMC7117761

[9] Wootton R. Recent advances: Telemedicine. BMJ. 2001;323: 557–560. doi: 10.1136/bmj.323.7312.557 11546704 PMC1121135

[10] Tuckson RV, Edmunds M, Hodgkins ML. Telehealth. N Engl J Med. 2017;377: 1585–1592. doi: 10.1056/NEJMsr1503323 29045204

[11] Wahezi SE, Kohan LR, Spektor B, Brancolini S, Emerick T, Fronterhouse JM, et al. Telemedicine and current clinical practice trends in the COVID-19 pandemic. Best Practice & Research Clinical Anaesthesiology. 2020; S1521689620301105. doi: 10.1016/j.bpa.2020.11.005 PMC7667401 34511221

[12] Hollander JE, Carr BG. Virtually Perfect? Telemedicine for Covid-19. N Engl J Med. 2020; NEJMp2003539. doi: 10.1056/NEJMp2003539 32160451

[13] Portnoy J, Waller M, Elliott T. Telemedicine in the Era of COVID-19. The Journal of Allergy and Clinical Immunology: In Practice. 2020;8: 1489–1491. doi: 10.1016/j.jaip.2020.03.008 32220575 PMC7104202

[14] Zhou X, Snoswell CL, Harding LE, Bambling M, Edirippulige S, Bai X, et al. The Role of Telehealth in Reducing the Mental Health Burden from COVID-19. Telemedicine and e-Health. 2020; tmj.2020.0068. doi: 10.1089/tmj.2020.0068 32202977

[15] Ankuda Claire K., Woodrell Christopher D., Meier Diane E., Morrison Sean, Chai Emily. A Beacon for Dark Times: Palliative Care Support During the Coronavirus Pandemic. NEJM Catalyst Innovations in Care Delivery. 2020. doi: 10.1056/CAT.20.0204

[16] Klein BC, Busis NA. COVID-19 is catalyzing the adoption of teleneurology. Neurology. 2020; 10.1212/WNL.0000000000009494. doi: 10.1212/WNL.0000000000009494 32238505

[17] Mehrotra A, Ray K, Brockmeyer DM, Barnett ML, Bender JA. Rapidly Converting to “Virtual Practices”: Outpatient Care in the Era of Covid-19. NEJM Catalyst Innovations in Care Delivery. 2020;1.

[18] Ohannessian R, Duong TA, Odone A. Global Telemedicine Implementation and Integration Within Health Systems to Fight the COVID-19 Pandemic: A Call to Action. JMIR Public Health Surveill. 2020;6: e18810. doi: 10.2196/18810 32238336 PMC7124951

[19] Smith AC, Thomas E, Snoswell CL, Haydon H, Mehrotra A, Clemensen J, et al. Telehealth for global emergencies: Implications for coronavirus disease 2019 (COVID-19). J Telemed Telecare. 2020; 1357633X2091656. doi: 10.1177/1357633X20916567 32196391 PMC7140977

[20] Calton B, Abedini N, Fratkin M. Telemedicine in the Time of Coronavirus. Journal of Pain and Symptom Management. 2020; S0885392420301706. doi: 10.1016/j.jpainsymman.2020.03.019 32240756 PMC7271287

[21] Bokolo AJ. Exploring the adoption of telemedicine and virtual software for care of outpatients during and after COVID-19 pandemic. Irish Journal of Medical Science (1971-). 2020; 1–10. 10.1007/s11845-020-02299-z PMC7340859 32642981

[22] Jnr BA. Use of telemedicine and virtual care for remote treatment in response to COVID-19 pandemic. Journal of Medical Systems. 2020;44: 1–9. 10.1007/s10916-020-01596-5 PMC7294764 32542571

[23] Jnr BA, Nweke LO, Al-Sharafi MA. Applying software-defined networking to support telemedicine health consultation during and post Covid-19 era. Health and technology. 2020; 1–9. 10.1007/s12553-020-00502-w PMC7605874 33163323

[24] Chmielewska B, Barratt I, Townsend R, Kalafat E, van der Meulen J, Gurol-Urganci I, et al. Effects of the COVID-19 pandemic on maternal and perinatal outcomes: a systematic review and meta-analysis. The Lancet Global Health. 2021; S2214109X21000796. doi: 10.1016/S2214-109X(21)00079-6 33811827 PMC8012052

[25] Allotey J, Stallings E, Bonet M, Yap M, Chatterjee S, Kew T, et al. Clinical manifestations, risk factors, and maternal and perinatal outcomes of coronavirus disease 2019 in pregnancy: living systematic review and meta-analysis. BMJ. 2020; m3320. doi: 10.1136/bmj.m3320 32873575 PMC7459193

[26] Mehrotra A, Nimgaonkar A, Richman B. Telemedicine and Medical Licensure—Potential Paths for Reform. N Engl J Med. 2021;384: 687–690. doi: 10.1056/NEJMp2031608 33626604

[27] Gunasekeran DV, Tham Y-C, Ting DSW, Tan GSW, Wong TY. Digital health during COVID-19: lessons from operationalising new models of care in ophthalmology. The Lancet Digital Health. 2021;3: e124–e134. doi: 10.1016/S2589-7500(20)30287-9 33509383

[28] Mansoor H, Khan SA, Afghani T, Assir MZ, Ali M, Khan WA. Utility of teleconsultation in accessing eye care in a developing country during COVID-19 pandemic. Liu Y-C, editor. PLoS ONE. 2021;16: e0245343. doi: 10.1371/journal.pone.0245343 33444381 PMC7808582

[29] Nicolás D, Coloma E, Pericàs JM. Alternatives to conventional hospitalisation that enhance health systems’ capacity to treat COVID-19. The Lancet Infectious Diseases. 2021;21: 591–593. doi: 10.1016/S1473-3099(21)00093-1 33711274 PMC8063075

[30] The Lancet. Facing up to long COVID. The Lancet. 2020;396: 1861. doi: 10.1016/S0140-6736(20)32662-3 33308453 PMC7834723

[31] Evans ML, Lindauer M, Farrell ME. A Pandemic within a Pandemic—Intimate Partner Violence during Covid-19. N Engl J Med. 2020;383: 2302–2304. doi: 10.1056/NEJMp2024046 32937063

[32] Chen Z, Chen L, Chen H. The impact of COVID-19 on the clinical trial. Samy AM, editor. PLoS ONE. 2021;16: e0251410. doi: 10.1371/journal.pone.0251410 33974651 PMC8112689

[33] Saúde M da. Coronavírus 2019: vigilância integrada de Síndromes Respiratórias. Brasília, DF: Ministério da Saúde Brasília; 2020 Abr. Available: https://www.saude.gov.br/images/pdf/2020/April/06/GuiaDeVigiEp-final.pdf

[34] Produto Interno Bruto dos Municípios | IBGE. [cited 20 May 2020]. Available: https://www.ibge.gov.br/estatisticas/economicas/contas-nacionais/9088-produto-interno-bruto-dos-municipios.html?=&t=o-que-e

[35] WordCloud for Python documentation—wordcloud 1.8.1 documentation. [cited 30 Dec 2020]. Available: https://amueller.github.io/word_cloud/

[36] Mikolov T, Sutskever I, Chen K, Corrado GS, Dean J. Distributed representations of words and phrases and their compositionality. Advances in neural information processing systems. 2013. pp. 3111–3119.

[37] Gensim: topic modelling for humans. [cited 30 Dec 2020]. Available: https://radimrehurek.com/gensim/auto_examples/index.html#documentation

[38] scikit-learn: machine learning in Python—scikit-learn 0.24.0 documentation. [cited 30 Dec 2020]. Available: https://scikit-learn.org/stable/

[39] Maaten L van der, Hinton G. Visualizing data using t-SNE. Journal of machine learning research. 2008;9: 2579–2605.

[40] Arons MM, Hatfield KM, Reddy SC, Kimball A, James A, Jacobs JR, et al. Presymptomatic SARS-CoV-2 Infections and Transmission in a Skilled Nursing Facility. N Engl J Med. 2020;382: 2081–2090. doi: 10.1056/NEJMoa2008457 32329971 PMC7200056

[41] Chen N, Zhou M, Dong X, Qu J, Gong F, Han Y, et al. Epidemiological and clinical characteristics of 99 cases of 2019 novel coronavirus pneumonia in Wuhan, China: a descriptive study. The Lancet. 2020;395: 507–513. doi: 10.1016/S0140-6736(20)30211-7 32007143 PMC7135076

[42] Hart JT. The inverse care law. The Lancet. 1971;297: 405–412. doi: 10.1016/s0140-6736(71)92410-x 4100731

[43] Else H. How a torrent of COVID science changed research publishing—in seven charts. Nature. 2020;588: 553–553. doi: 10.1038/d41586-020-03564-y 33328621

[44] Castro MC, Resende de Carvalho L, Chin T, Kahn R, Franca GVA, Macario EM, et al. Demand for hospitalization services for COVID-19 patients in Brazil. Infectious Diseases (except HIV/AIDS); 2020 Apr. doi: 10.1101/2020.03.30.20047662

[45] Onder G, Rezza G, Brusaferro S. Case-Fatality Rate and Characteristics of Patients Dying in Relation to COVID-19 in Italy. JAMA. 2020 [cited 29 May 2020]. doi: 10.1001/jama.2020.4683 32203977

[46] Guan W, Ni Z, Hu Y, Liang W, Ou C, He J, et al. Clinical Characteristics of Coronavirus Disease 2019 in China. N Engl J Med. 2020; NEJMoa2002032. doi: 10.1056/NEJMoa2002032 32109013 PMC7092819

[47] Abate SM, Ahmed Ali S, Mantfardo B, Basu B. Rate of Intensive Care Unit admission and outcomes among patients with coronavirus: A systematic review and Meta-analysis. Lazzeri C, editor. PLoS ONE. 2020;15: e0235653. doi: 10.1371/journal.pone.0235653 32649661 PMC7351172

[48] Mohammed MA, Clements G, Edwards E, Lester H. Factors which influence the length of an out-of-hours telephone consultation in primary care: a retrospective database study. BMC health services research. 2012;12: 430. doi: 10.1186/1472-6963-12-430 23181707 PMC3542015

[49] Bai Y, Yao L, Wei T, Tian F, Jin D-Y, Chen L, et al. Presumed Asymptomatic Carrier Transmission of COVID-19. JAMA. 2020;323: 1406. doi: 10.1001/jama.2020.2565 32083643 PMC7042844

[50] Kimball A, Hatfield KM, Arons M, James A, Taylor J, Spicer K, et al. Asymptomatic and Presymptomatic SARS-CoV-2 Infections in Residents of a Long-Term Care Skilled Nursing Facility—King County, Washington, March 2020. MMWR Morb Mortal Wkly Rep. 2020;69: 377–381. doi: 10.15585/mmwr.mm6913e1 32240128 PMC7119514

[51] Chiu C-Y, Sarwal A, Jawed M, Chemarthi VS, Shabarek N. Telemedicine experience of NYC Internal Medicine residents during COVID-19 pandemic. Rowley JA, editor. PLoS ONE. 2021;16: e0246762. doi: 10.1371/journal.pone.0246762 33556151 PMC7869991

[52] Processo No: 33910.007111/2020-95 Nota Técnica No 4/2020/GGRAS/DIRAD-DIPRO/DIPRO. Agência Nacional de Saúde; Available: https://www.ans.gov.br/images/stories/noticias/pdf/covid_19/nota-tecnica-4-2020-ggras-dirad-dipro-dipro.pdf

[53] Silva MA, Perez OFR, Añez LM, Paris M. Telehealth treatment engagement with Latinx populations during the COVID-19 pandemic. The Lancet Psychiatry. 2021;8: 176–178. doi: 10.1016/S2215-0366(20)30419-3 33038976 PMC7544483

